# Aluminium Nitride Surface Characterization by Grinding with Laser–Ultrasonic Coupling

**DOI:** 10.3390/ma17153772

**Published:** 2024-08-01

**Authors:** He Zhang, Cong Sun, Yuan Hong, Yansheng Deng, Liang Ma

**Affiliations:** 1 School of Mechanical Engineering and Automation, Northeastern University, Shenyang 110819, China; 2270273@stu.neu.edu.cn (H.Z.); 2110112@stu.neu.edu.cn (Y.H.); 2 National Institute of Intelligent Robotics Shenyang Co., Ltd., Shenyang 110015, China; 3 Jihua Laboratory, Foshan 528251, China; dengys@jihualab.com; 4 School of Mechanical and Electrical Engineering, Xinjiang Institute of Engineering, Urumqi 830023, China; maliang7653@sina.com

**Keywords:** aluminium nitride, laser–ultrasonic-assisted grinding, molecular dynamics, surface hardness, grinding force

## Abstract

Aluminium nitride (AlN) materials are widely used in heat-dissipation substrates and electronic device packages. However, the application of aluminium nitride ceramics is hindered by the obvious anisotropy and high brittleness of its crystals, leading to poor material surface integrity and high grinding force. With the rapid development of microelectronics, the requirements for the material’s dimensional accuracy, machining efficiency, and surface accuracy are increasing. Therefore, a new machining process is proposed, combining laser and ultrasonic vibration with grinding. The laser–ultrasonic-assisted grinding (LUAG) of aluminium nitride is simulated by molecular dynamics (MD). Meanwhile, the effects of different processing techniques on grinding force, stress distribution, matrix damage mechanism, and subsurface damage depth are systematically investigated and verified by experiments. The results show that laser–ultrasonic-assisted grinding produces 50% lower grinding forces compared to traditional grinding (TG). The microhardness of AlN can reach more than 1200 HV, and the coefficient of friction and wear is reduced by 42.6%. The dislocation lines of the AlN substrate under this process are short but interlaced, making the material prone to phase transformation. Moreover, the subsurface damage depth is low, realising the substrate’s material hardening and wear resistance. These studies not only enhance the comprehension of material build-up and stress damage under the synergistic impact of laser, ultrasonic, and abrasive processing but also indicate that the proposed method can facilitate and realise high-performance machining of aluminium nitride substrate surfaces.

## 1. Introduction

Aluminium nitride (AlN) crystals are an excellent encapsulation material in microelectronics due to their properties such as high thermal conductivity, high dielectric coefficient, reliable electrical insulation, and thermal expansion coefficient matching silicon [1]. However, brittle fractures generated during the fabrication process can significantly reduce the application accuracy and service life of AlN ceramics [2]. For brittle materials, abrasive grain processing methods such as grinding [3] and polishing [4] are effective means to achieve ultra-smooth surfaces with nanoscale roughness [5,6,7]. 

Gao et al. [8] built a molecular dynamics model of single-crystalline AlN scratches. They determined that plastic deformation is realised by basal and conical dislocations, shifting, and horizontally expanding laminar dislocations. When the scratch depth exceeds 6 nm, the brittle fracture of the material occurs on the disintegration surface of single-crystal AlN. Cai et al. [9] conducted variable-force single-scratch and constant-force double-scratch tests on AlN ceramics. The results showed that under cumulative loading, the increase in stress caused the material to undergo a brittle-to-plastic transition. In order to minimise the effect of brittle deformation on the material, Gao et al. [10] conducted constant-force single-scratch tests on AlN ceramics with different scratching speeds. They found that higher strain rates are favourable for the plastic flow of AlN ceramics. Li et al. [11] concluded that the critical grinding depth of AlN ceramics for the toughness and brittle transition is 140–240 nm through variable-load nano-scratching experiments. Although the AlN single-crystal grinding process realises a nanoscale ultra-smooth grinding surface, a damage layer hundreds of nanometres deep on its subsurface still exists. There is serious plastic and brittle damage in the local grinding region. Meanwhile, the traditional grinding method is time-consuming, costly, and inflexible. For AlN ceramic materials with low fracture toughness, reducing the subsurface damage and improving the surface quality have become urgent problems. Therefore, exploring new hybrid machining methods to improve machinability is necessary. For hard and brittle, difficult-to-machine materials, there are many non-traditional machining methods, including laser-assisted grinding (LAG) [12,13] and ultrasonic vibration-assisted grinding (UVAG) [14,15]. These machining methods have been demonstrated to improve surface quality and reduce cutting forces [16]. Li et al. [17] performed molecular dynamics (MD) simulations of the LAG process of gallium nitride single crystals by LAMMPS (Large-Scale Atomic/Molecular Parallel Simulator). The study’s results enhance the understanding of material removal and damage under the coupled action of laser and abrasive processing and provide a theoretical basis for parameter optimisation during GaN single-crystal processing. Ma et al. [18,19] obtained the simulated grinding force distribution on the workpiece surface under different laser powers by modelling. The results showed that higher laser power improves surface integrity and reduces damage depth within a certain threshold. Li et al. [20] found that the structural changes and hardness reduction induced by LAG improved the plasticity removal effect. This method effectively improved the surface quality and reduced the depth of subsurface damage as compared to TG. Chen et al. [21] conducted UVAG tests on sapphire crystals. They found that the UVAG technique effectively reduces the grinding force and specific grinding energy compared with TG. Zhang et al. [22] conducted ultrasonic vibration scratching experiments on sapphire using a spherical diamond indenter. The experimental results show that the ultrasonic-assisted machining process can reduce the scratch load, effectively inhibit microcrack extension, and increase the plasticity removal ratio. Zhao et al. [23] revealed the surface formation mechanism of ultrasonic elliptical vibration-assisted diamond cutting single-crystal 3C-SiC machining through molecular dynamics simulation. The ultrasonic elliptical vibration-assisted diamond cutting has a significant effect of reducing cutting force, inhibiting cracks, and promoting brittle material cutting. Suarez et al. [24] examined the surface integrity, hardness, residual stress, and fatigue strength obtained from these machining processes for cutting alloy 718, showing an improvement in the surface integrity of the final workpiece. From the above study, the advantages of UVAG and LAG have been verified, but few studies have been conducted on the hybrid machining of LAG and UVAG [25]. Meanwhile, few reports are on the simultaneous application of LAG and UVAG to the machining of AlN ceramics.

In order to maximise the mechanical properties of AlN ceramics in the grinding process while reducing surface defects and stress damage during machining, a laser–ultrasound-assisted grinding method for machining of AlN ceramics is proposed. MD simulations of traditional grinding, laser-assisted grinding, ultrasonic-assisted grinding, and laser–ultrasonic-assisted grinding of aluminium nitride are carried out. The grinding force, chip build-up increases, dislocation density, stress distribution, phase transformation process, and subsurface damage under different working conditions are systematically studied by the visualisation software OVITO (Open Visualization Tool Version 3.84). The laser heating not only reduces the grinding force but also increases the density of dislocations and realises the hardening of the machined surface. The axial motion of abrasive particles under ultrasonic action can significantly reduce the roughness of the machined surface, considerably reduce the generation of dislocations, and improve the damage to the subsurface. These studies provide a theoretical basis for optimising experimental data in the high-performance processing of aluminium nitride, broadening the field of research on improving the mechanical properties of aluminium nitride by coupling effects.

## 2. Modelling on the Molecular Dynamics of LUAG

LAMMPS enables efficient parallel analysis of structural phase transitions and material defects resulting from interatomic interactions. The savings of experimental resources are realised by the repeated simulation optimisation. Thereafter, the visualisation software OVITO is used to numerically calculate the simulation results and render the image output.

### 2.1. MD Simulation for Single Abrasive Grinding on Aluminium Nitride

**Figure 1:**
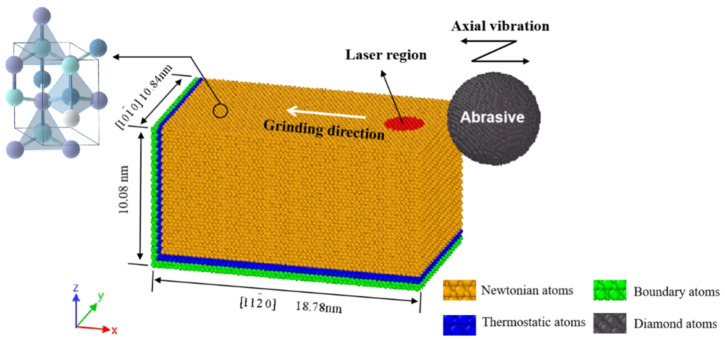
Three-dimensional MD simulation model.

Due to the complex interactions between the workpiece and the abrasive particles, single-grain grinding is usually used to investigate the material processing and damage mechanism during the grinding process. Therefore, in this section, MD simulation is carried out on single abrasive-assisted grinding under laser and ultrasonic coupling effects. Meanwhile, AlN’s crystal defects and stress damage evolution mechanism will be explored. As shown in Figure 1, the three-dimensional MD simulation model is simulated by LAMMPS (Version 2Aug2023) software. The aluminium nitride tetrahedron consists of one aluminium atom and four neighbourhood N atoms. The three-dimensional MD simulation model has boundary dimensions of 18.8, 10.8, and 10.1 nm, respectively. To preserve the model’s integrity, the thickness and thermostatic layer of the boundary are established as an integer multiple of the lattice dimension. $\left[11\bar{2}0\right]$ is selected as the direction of grinding, which is conducive to achieving plastic deformation [26]. To improve the computational efficiency, the abrasive model is a rigid spherical configuration which does not rotate. For MD simulations, the Vashishta potential function, widely used to predict cohesive energy in aluminium nitride, has been selected. This function accurately depicts the material characteristics and phase transitions in the machining of AlN ceramics, particularly in terms of crack generation and propagation within the brittle regime. The mathematical expression for this function is provided as follows [27]:

(1) $$V=\sum_{i}^{N}\sum_{j>i}^{N}V_{ij}^{\left(2\right)}(r_{ij})+\sum_{i}^{N}\sum_{j\neq i}^{N}\sum_{k>j,k\neq i}^{N}V_{ijk}^{\left(3\right)}(r_{ij},r_{ik},\theta_{ijk})$$

(2) $$V_{ij}^{\left(2\right)}(r)=\;\frac{H_{ij}}{r^{n_{ij}}}+\frac{Z_{i}Z_{j}}{r}e^{-r/\lambda }-\frac{D_{ij}}{r^{4}}e^{-r/\xi }-\frac{W_{ij}}{r^{6}},r<r_{c,ij}$$

(3) $$V_{ijk}^{\left(3\right)}(r_{ij},r_{ik},\theta_{ijk})=B_{ijk}\frac{{\left[\cos\theta_{ijk}-\cos\theta_{0ijk}\right]}^{2}}{1+C_{ijk}{\left[\cos\theta_{ijk}-\cos\theta_{0ijk}\right]}^{2}}\times e^{\left(\frac{r_{ij}}{r_{ij}-r_{0,ij}}\right)}\times e^{\left(\frac{r_{ik}}{r_{ik}-r_{0,ik}}\right)},r_{ij}<r_{C,ij},r_{ik}<r_{0,ik}$$

where $H_{ij}$ and $n_{ij}$ are the strength and exponent of the repulsive force; $W_{ij}$ and $D_{ij}$ represent the van der Waals force and the charge dipole strength, respectively. $Z_{i}$ denotes the effective charge in terms of charge |e|; *ξ* and *λ* represent the shielding length of the charge dipole and the coulombic force, respectively. $r_{ij}$ is the distance between the two atoms *i* and *j*, *B* is the strength of the three-body interaction, $r_{0}$ is the truncation radius, *θ_ijk_* is the angle formed by $r_{ij}$ and $r_{ik}$, and *θ*_0*ijk*_ and *C_ijk_* are constants. In addition, the interaction potential between the diamond abrasive grains and the workpiece atoms is adopted as the Lennard-Jones potential, with the truncation radius chosen as 10 Å. The interatomic potential function of the diamond abrasive grains is adopted as the Tersoff potential function.

### 2.2. Establishment of Multi-Field Processing Conditions

In the simulation of a single-grain LUAG process, only the thermal effect of the laser is taken into account. Compared to the “fix heat” command, the thermal effect of the laser is realised by the “fix ehex” procedure, which precisely reduces the thermal drift. The heating area of the laser is a cylinder with the diameter and height of 2 nm. The laser temperature is chosen at 800 K to ensure the material undergoes sufficient phase transformation while avoiding atom escape. Meanwhile, to increase grinding efficiency and improve surface quality, the grinding trajectory is axial vibration. The velocity trajectory of the diamond abrasive is as follows [28]:

(4) $$\left\{\begin{array}{l} v_{x}=A\sin(2\pi \frac{1}{T}t)\\ v_{z}=0 \end{array}\right.$$

where *v_x_* and *v_z_* are the abrasive velocities along the X and Z axes, *A* is the vibration amplitude, and *T* is the vibration period. The simulation parameters are shown in Table 1.

## 3. Results and Discussions

### 3.1. RDF Analysis of AlN Structure

**Figure 2:**
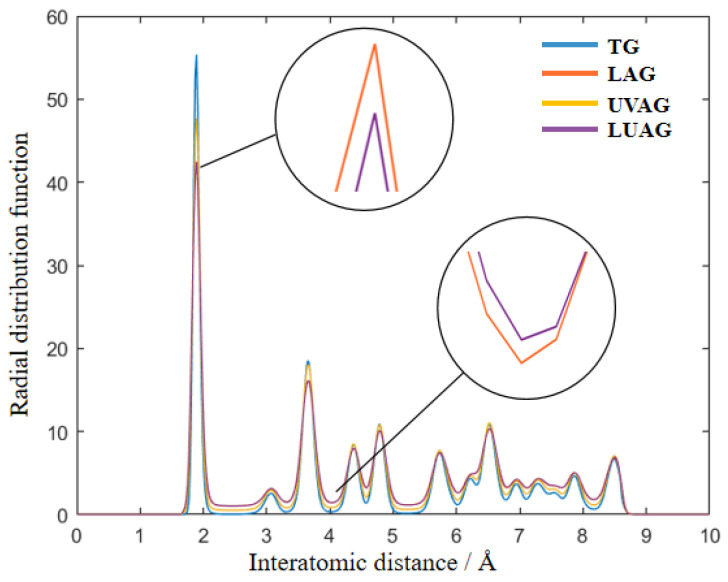
The RDF of Al–N, Al–Al, and N–N bonds of AlN via the four processing technologies.

The radial distribution function (RDF) is generally applied to the analysis of physical parameters of structures in MD. The structural characteristics and reaction behaviour of materials can be revealed by describing the spatial distribution of atoms and molecules. Meanwhile, the peaks’ position, shape, and periodicity can be used to determine the crystalline and amorphous structural changes. As shown in Figure 2, the atomic spacing of Al-N, N-N/Al-Al, N-Al, and Al-Al/N-N is 1.90 Å, 3.11 Å, 3.64 Å, and 4.37 Å, respectively. The crystal lattice of the aluminium nitride crystals is relatively intact under TG. However, with the different auxiliary machining processes, the peaks of the RDF of aluminium nitride become flatter. The RDF curve incorporating the laser-assisted process is conspicuous and most prominent in its peak shape and value. Compared with the mechanical action of abrasive grains during the grinding process, thermal stress is more conducive to prompting the phase transition. Therefore, the atomic structure is less stable and easier to remove. Among them, the LUAG has the flattest and widest peak. Thus, it is concluded that aluminium nitride atoms are more likely to undergo lattice deformation and structural phase transformation under LUAG.

### 3.2. Analysis of the Grinding Force and Chip Build-Up Increases

**Figure 3:**
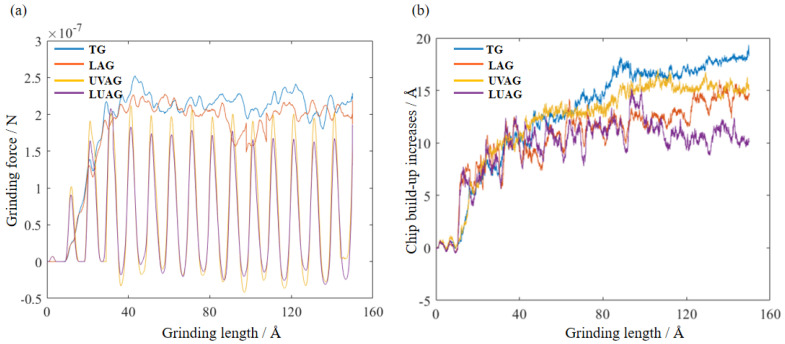
The grinding force and the amount of growth via the four processing technologies. (**a**) The grinding force (**b**) The grinding force.

The force field between diamond atoms and aluminium nitride atoms is simulated by LAMMPS. The curve of four processes on the grinding force is derived as shown in Figure 3a. In the diagram, TG has the highest forces and LUAG has the most minor forces. To avoid atom escape, the main effect of the laser is making the material softer rather than material removal. As shown in Figure 3b, chip build-up in the grinding area is reduced as laser heating helps to improve chip flow and discharge. Therefore, the grinding force in this process is conspicuously smaller than in TG. The UVAG can obviously improve the fluidity of the material atoms and reduce the friction coefficient. Consequently, there is difficulty in the chip adhering to abrasive surfaces, which effectively suppresses chip build-up and reduces grinding forces compared to TG and LAG. Meanwhile, the grinding force can be negative during ultrasonic-assisted grinding because the force field attracts the diamond and aluminium nitride atoms to each other. Laser heating reduces the hardness and brittleness of the material, while ultrasonic vibration promotes chip breakage and expulsion. Therefore, LUAG requires less energy to remove a large amount of graphite-like cubic aluminium nitride produced by laser heating. The grinding force is less than that of conventional grinding, which destroys the atomic structure of a closely packed hexagonal aluminium nitride.

### 3.3. Analysis of the Atom Flow Field and Von Mises Shear Stress

**Figure 4:**
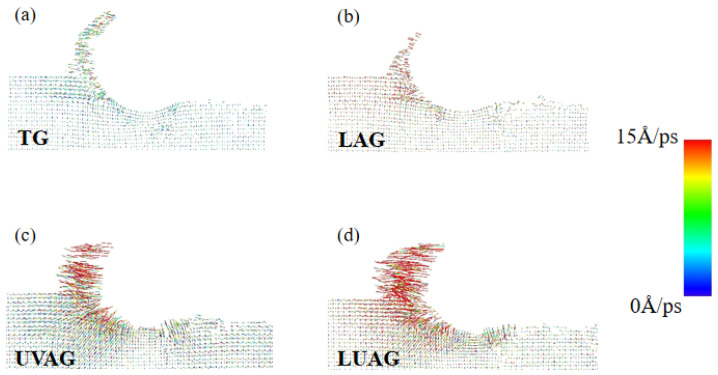
The atom flow field via the four processing technologies. (**a**) TG (**b**) LAG (**c**) UVAG (**d**) LUAG.

The atomic flow field is one of the compelling methods for studying substrate materials in nano-grinding. Figure 4a–d show the flow velocity of atoms under distinct processing conditions. Compared with Figure 4a, the thermal effect of the laser and the axial vibration of abrasive particles in Figure 4b and Figure 4c both improve the dynamic mobility of chip atoms. In UVAG, the persistent “impinging” of the abrasive grain on the aluminium nitride substrate improves the dynamic mobility of the atoms more significantly. As shown in Figure 4d, LUAG causes high-frequency vibrations of atoms near their equilibrium positions, which enhances the interaction force between atoms. Therefore, the flow rate is increased in the grinding zone, and the length of the atomic trajectory line places the motion of the aluminium nitride atom in a malleable state. Grinding under ductility has a positive effect on the material removal and AlN mechanical properties.

**Figure 5:**
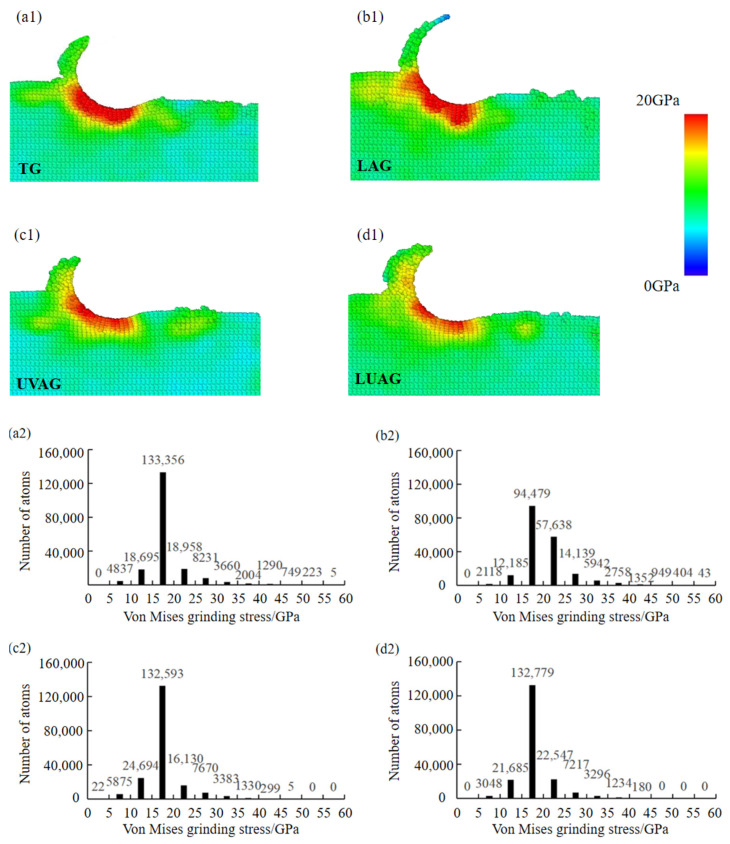
The von Mises shear stress via the four processing technologies. (**a1**–**a2**) TG (**b1**–**b2**) LAG (**c1**–**c2**) UVAG (**d1**–**d2**) LUAG.

The equivalent von Mises stress equation is a yield criterion applied to identify the yield stress state [29] and the stress is expressed as

(5) $$\sigma =\frac{1}{\sqrt{2}}{\left[{\left(\sigma_{1}-\sigma_{2}\right)}^{2}+{\left(\sigma_{2}-\sigma_{3}\right)}^{2}+{\left(\sigma_{3}-\sigma_{1}\right)}^{2}\right]}^{1/2}$$

where $\sigma_{1}$, $\sigma_{2}$, and $\sigma_{3}$ are the principal stress. Figure 5a1–d1 show the cross-sectional contours of von Mises shear stresses for different processes. The bar graphs in Figure 5a2–d2 show that the values of TG stresses and LAG stresses are larger. The maximum values of stress in TG and LAG conditions are close to 60 GPa. On the contrary, the number of atoms in the high-pressure region in UVAG and LUAG conditions are zero and concentrated in the 15–20 GPa interval. This is due to the fact that LAG reduces the interatomic interaction forces, softens the material, and increases the removal rate of the material in the range of the interval of warming up to 800K. Therefore, the mechanical stresses generated by grinding are reduced. It is worth noting that in order to ensure surface roughness, laser heating does not directly remove the surface material. The reduction in mechanical stress is limited, resulting in superimposed mechanical stress and thermal stress that are slightly higher than that of TG. Due to UVAG accelerating the displacement of chip atoms, the stored energy of chip atoms increases while reducing the residual stresses on the machined surface. The stress distribution under UVAG is more uniform and the stress layer is thinner. Residual stresses due to grinding forces are released and dissipated under the coupling effect of LUAG. Therefore, compared with a single processing method, LUAG reduces mechanical stress by softening the material while reducing the accumulation and concentration of stresses within the material. Meanwhile, LUAG has a longer displacement of chip atoms; more chip atoms are removed and the energy share of chip atoms is larger. Accordingly, the residual stress on the surface of the substrate under the composite energy field is significantly reduced, and the surface quality of the substrate is improved.

### 3.4. Analysis of the Subsurface Damage Depth

**Figure 6:**
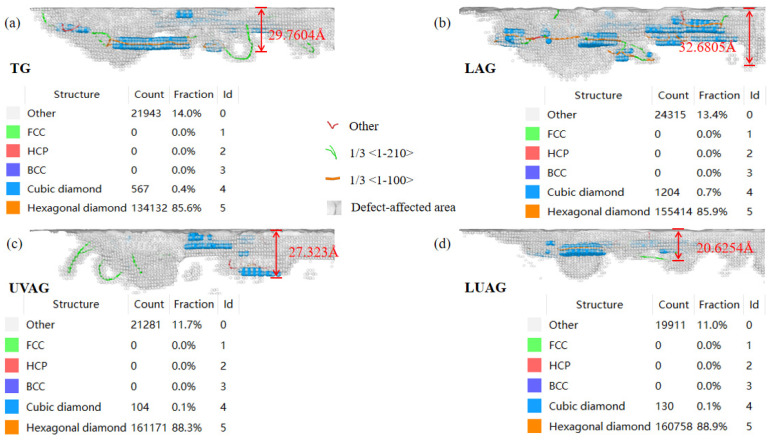
The subsurface damage depth via the four processing technologies. (**a**) TG (**b**) LAG (**c**) UVAG (**d**) LUAG.

Figure 6 shows the subsurface damage depth by different processes. The blue atomic depth of the phase transition is the damage depth and the grey area is chosen as the defect-affected area. When the crystal cells reach the critical stress and phase transition temperature by the high temperature and high pressure of LAG, the Al and N atoms undergo intense thermal motion. Therefore, the atomic arrangement and lattice structure of the material change. Some of the densely arranged hexagonal aluminium nitride phases become graphite-like cubic aluminium nitride and a small number of amorphous bodies. As the laser beam irradiation to the material surface leads to localised high temperatures, the thermal stress concentration inside the material produces microcracks or crack extensions. Therefore, coupling mechanical and thermal stresses under LAG conditions causes more damage to the subsurface layer than TG. UVAG primarily relies on the phase transition triggered by high pressure, and the surface damage brought about by the grinding process is reduced by repeated ironing as well as pressing on the surface of the substrate material. Meanwhile, the vibration effect helps to promote the discharge of grinding chips and reduce the clogging phenomenon in the grinding process, thus reducing the damage to the aluminium nitride subsurface. It can be clearly seen that the depth of subsurface damage is minimised under LUAG in Figure 6d. This is because the stability of graphite-like cubic aluminium nitride after phase transformation is lower than that of closely packed hexagonal aluminium nitride, and the softening of the material is more conducive to the removal [30,31,32]. From the results in Figure 3, LUAG optimises the trajectory of the abrasive particles and the distribution of grinding forces during the grinding process, reducing the proportion of brittle removal. Therefore, the coupling reduces the occurrence of subsurface damage.

### 3.5. Analysis of the Dislocation Properties

**Figure 7:**
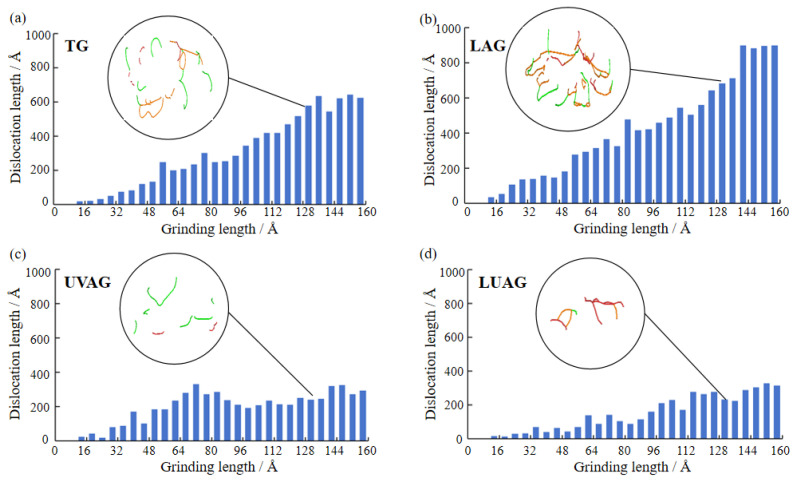
The surface mechanical properties via the four processing technologies. (**a**) TG (**b**) LAG (**c**) UVAG (**d**) LUAG.

The histograms and morphology of dislocation lines are shown in Figure 7a–d under the four processes. The material is an intact crystal with great strength in the face of small dislocation density. The material undergoes work hardening under high dislocation density. The laser’s thermal stress promotes dislocation slip, so the dislocation density is significantly higher than that of TG [33,34,35]. With the increase in thermal stress, the increase in cubic aluminium nitride after phase transformation hinders the slippage of the dislocation line. Meanwhile, the generation of residual stresses leads to the formation of dislocation lines and an increase in local density, as shown in Figure 7b. UVAG improves lattice distortion by increasing the mobility of atoms. The high mobility of atoms provides a greater migration driving force for dislocations, which merge or disappear between dislocations. From Figure 7c, the significant decrease in the number of dislocations, along with their dispersed and ductile arrangement, is evident. In the LUAG process, the laser heat can propagate uniformly inside the aluminium nitride material under ultrasound, resulting in a more uniform energy distribution. This uniform energy distribution reduces the high number of dislocation lines caused by localised high temperatures or high stresses. In Figure 7d, the migration capacity of aluminium nitride atoms is significantly improved when laser and ultrasonic coupling is used. The overall length of dislocations is conspicuously reduced compared with the rest of the process under the diamond grit, but the distribution of dislocations is characterised by interlacing, improving the microhardness of the material.

## 4. Experimental Characteristics and Validation

**Figure 8:**
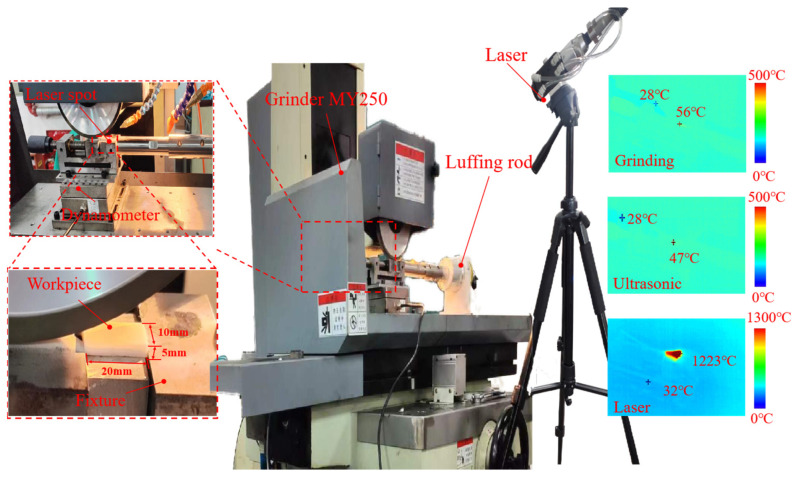
LUAG experimental platform.

As shown in Figure 8, the experimental platform is built based on an automatic surface grinder (MY250) containing a grinding force measurement system, a laser module, and an ultrasonic module. The grinding wheel used for processing aluminium nitride ceramics is a diamond wheel with a diameter of 180 mm and a width of 13 mm. The grit size of the grinding wheel is 320#. The experimental material for this study is a typical structural ceramic, aluminium nitride, with a purity of 99%, a particle size of less than 1.5 microns, and workpiece size of 2 × 1 × 0.5 cm. Aluminium nitride ceramics have excellent mechanical strength and thermal shock resistance and are chemically stable. The NC-DAQX dynamometer dynamically monitors the dynamic grinding forces generated during the machining process in three directions. The sensors convert the grinding forces into electrical signals, which are enhanced by an amplifier and stored in the acquisition module. Finally, the measured data are transferred to a computer for filtering and dynamic visualisation. In order to ensure the roughness of the surface of the processed material, laser heating at 500 (J/s) after several comparisons is used. Vibration is transmitted to the workpiece on the fixture by means of a luffing rod. The equipment used in this experiment is Hang Zhou Guo Biao ultrasonic equipment; the amplitude range of the equipment is 0–10 microns. The appropriate amplitude can effectively reduce the surface roughness while improving the material removal efficiency. Therefore, the ultrasonic amplitude is chosen as 8 µm in this experiment. Meanwhile, the frequency of the equipment is set to 20 KHz to ensure ultrasonic conditions. Laser-assisted machining involves abrasive materials grinding the surface of a laser-irradiated workpiece, and there is a certain positional difference between the abrasive material and the laser. Therefore, the temperature contours after the grinding process, the ultrasonic-assisted grinding process, and laser irradiation are measured separately by a UTI640J (UNI-T (China) Co., Ltd., Changzhou, China) focusable infrared thermal imager, as shown in Figure 8. It can be concluded that ultrasonic vibration is beneficial in reducing grinding heat, and at the same time, the laser power of 500 J/s can cause structural phase transformation of the material. Meanwhile, no lubricant is used in the experimental process because aluminium nitride reacts spontaneously with water in a hydrolysis manner. According to previous studies, the grinding experimental conditions are shown in Table 2.

### 4.1. Microhardness and Surface Roughness of the AlN

**Figure 9:**
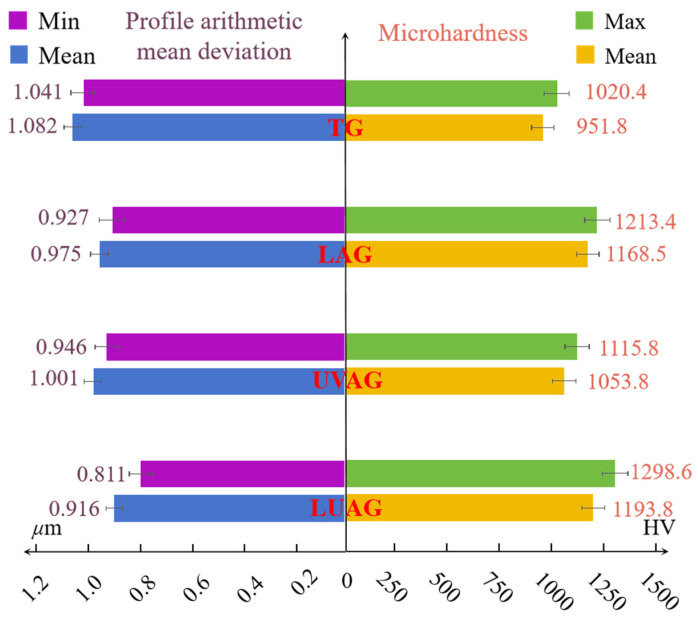
Microhardness and surface roughness of the machining area.

Figure 9 shows a comparison of the surface roughness and the histogram of microhardness under different processes. Mean value selection can effectively reduce the random error and improve the measurement’s accuracy and reliability. Meanwhile, a more comprehensive response to the overall mechanical properties of the material surface can lead to further improvements. Therefore, hardness measurements are carried out at ten randomly selected equally spaced measurement points within the grinding zone of the aluminium nitride ceramic surface. The final surface hardness is expressed by averaging the ten measurements. An HV-1000Z (Shanghai JOHOYD, Shanghai, China) microhardness tester is commonly used for microhardness testing of thin and small specimens and brittle materials. Therefore, the tester is used for the experiment to compare the strengthening effect of different auxiliary processes on the surface of aluminium nitride. The microhardness of the laser–ultrasonic composite is slightly higher than that of laser-assisted grinding. LUAG enables more uniform and efficient material removal by coupling a laser and ultrasound. This coupling helps to create a more homogeneous and dense surface structure. Meanwhile, promoting the efficient accumulation of dislocation lines thus increases the surface hardness of the aluminium nitride material. Compared to TG, its hardness increases by 26.7%, with 1298.6 HV at the highest point of hardness. Compared with the ultrasonic vibration action, laser heating can strengthen the AlN surface more conspicuously. The plastic flow of the material in the presence of multiple fields is significantly improved. The plastic flow, accompanied by the enhanced oxidation reaction, improves aluminium nitride’s surface damage. Using the Time-3230 (Beijing Times Guangnan Detection Technology Co., Ltd., Beijing, China) for surface roughness measurement, the sampling length selected is 0.8 mm. The surface roughness is compared by Ra (Profile Arithmetic Mean Deviation). Compared to TG, the surface roughness under LUAG was reduced by 28.4%. The surface roughness of the substrate after LUAG is minimised without obvious scratches, which is in accordance with the theory about dislocation lines and phase transitions mentioned before.

### 4.2. Grinding Forces during Machining

**Figure 10:**
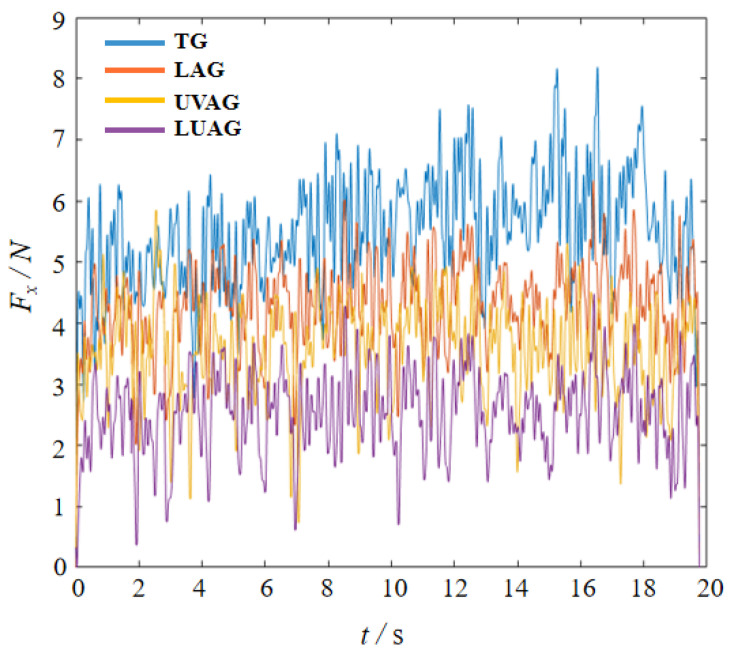
Measurement results of dynamic grinding force.

The effect of different machining processes on the grinding feeding force under the machining varied, with LUAG having the most prominent effect on reducing the grinding force. Figure 10 shows a dynamic plot of the grinding force over time during machining. The composite energy field reduced the grinding force by 50%. This is due to the structural phase transition of the material caused by laser heating during the machining process. The static equilibrium volume of the cubic structure is reduced after the phase transition, which is macroscopically manifested as the softening of the material. At the same time, the axial vibration of the abrasive grain under UVAG improves the fluidity of the chips, making it difficult for them to accumulate during the grinding process. Therefore, the contact area between the abrasive grits and the material is smaller under UVAG than under TG, and the grinding force is consequently reduced. The alumina generated during the grinding process acts as a solid lubricant, reducing energy loss and thus facilitating the flow and removal of atoms. The measured results in Figure 10 have the same tendency as the simulation in Figure 3 for single abrasive grinding force, stating the rationality of the analysis regulation.

### 4.3. Friction and Wear Characteristics of Machined Surfaces

**Figure 11:**
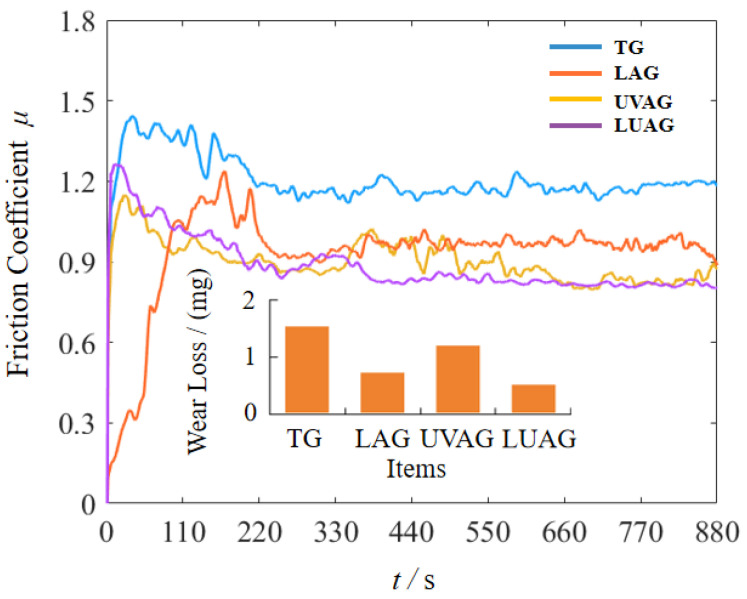
Statistics of friction and wear test results.

The DSR-II tribological and abrasion testing machine is used for friction and wear tests. Due to specific tiny grooves on the surface of aluminium nitride after machining, the contact area with the alumina ceramic ball is not ideal. Therefore, the friction wear curve shows an increasing trend and apparent fluctuation when the ceramic ball contacts the material surface, as shown in Figure 11. With the continuous friction between the ceramic ball and the surface, the tiny grooves are gradually removed, thus forming a new contact surface. Afterwards, the friction wear stabilises by deliquescence of the aluminium nitride to produce the “solid lubricant” aluminium oxide. Under the effect of the thermal stress of the laser, there are significant fluctuations in the laser-assisted grinding process. The aluminium nitride matrix’s subsurface is characterised by localised stress concentrations and microscopic crevices. Due to the concentration of thermal stresses on the aluminium nitride subsurface by a single laser, certain material defects are created. As a result, these stress damages and material structure deformations increase the friction wear coefficient of the LAG. However, as the ceramic ball is continuously rubbed, some of the damaged areas of the material are removed and ameliorated, and the friction wear curve thus decreases and gradually smooths out. The friction wear rate is defined as [37]

(6) $$W=\frac{V}{F\times L}$$

where *V* is the wear volume, *F* is the friction load, and *L* is the wear length. LUAG can effectively improve the mechanical properties of aluminium nitride surfaces with smaller values and stable trends in friction and wear curves. Compared to TG, the friction coefficient of the aluminium nitride surface under LUAG coupling decreased by 42.6%. This indicates that LUAG increases the strength and improves the friction and wear performance of the surface material.

### 4.4. X-ray Diffraction (XRD) Spectrum Analysis

**Figure 12:**
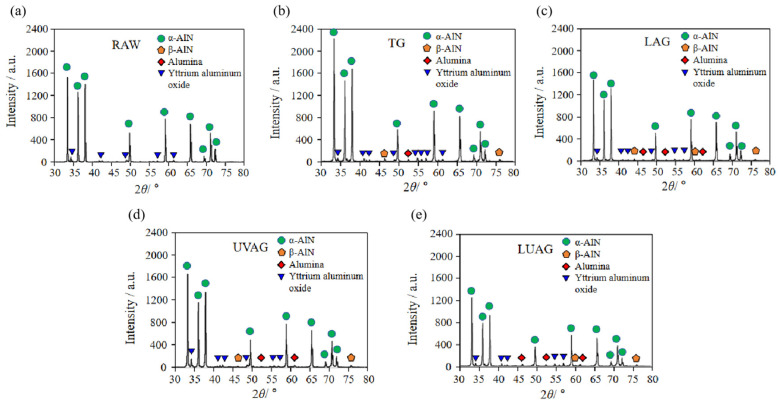
X-ray diffraction (XRD) spectrum of AlN ceramics. (**a**) Raw surface (**b**) TG surface (**c**) LAG surface (**d**) UVAG surface (**e**) LUAG surface.

In order to prove the phase transition characteristics of aluminium nitride, the experiment is examined utilising the Rigaku Smartlab. For this purpose, a Cu-Kα1 radiation anode is employed to scan the samples in increments of 0.02°. Within a 2θ angle range spanning from 10° to 90°, we have selected the most representative range of 30° to 80°. XRD analysis is carried out on the base material and the workpieces processed by four processes, respectively. Aluminium nitride, under high temperature or pressure, produces a structural phase transition, which is the cubic structure of aluminium nitride. As shown in Figure 12a–e, through the XRD analysis, it can be concluded that compared with the unprocessed base material, the processed aluminium nitride ceramics have undergone a certain degree of structural phase transition. Among them, the cubic aluminium nitride structural term is the most obvious after laser irradiation. In addition, the processed ceramic matrix contains an alumina phase resulting from the oxidation reaction [38] and an impurity phase of yttrium aluminium oxide, which is an additive during the preparation process.

## 5. Conclusions

To fill the knowledge gap of the interaction of coupled-field effects with phase transitions in aluminium nitride structures, a new process for machining aluminium nitride is proposed. The effects of different machining conditions on the surface quality, structural phase transition, chip morphology, and grinding force of aluminium nitride are discussed by comparing experiments and MD simulations. The following conclusions can be drawn:

The combined application of laser and ultrasonic technologies enhances the temperature of the workpiece region in front of the grinding wheel, which generates a homogeneous heat-affected zone. This thermal motion leads to a decrease in the bond strength of the atoms and the stability of the crystal structure, resulting in softening and a decrease in the hardness of the material. Ultrasonic refinement allows the material to be refined while avoiding chip build-up by improving the flow of chips. This coupling effect diminishes the grinding force by nearly 50%.

- The structural phase change and dislocation migration occurred in all materials when aluminium nitride is processed via grinding. Among them, the aluminium nitride under the LUAG process has the smallest subsurface affected zone and the shortest total length of dislocation lines. As a result, the hardness of the aluminium nitride surface affected by the coupling effect can reach 1236.9 HV, which is 26.7% harder than TG. The machined surface quality was significantly improved under this process, and the material’s friction coefficient was reduced by nearly 42.6%.
- LUAG makes the surface of aluminium nitride have a better stress state. The residual stress layer on the machined surface is smaller and uniform, while the stress in the affected area in front of the abrasive grain during the grinding process is obviously lower than that in the conventional grinding process.
- LUAG enables ductility removal of aluminium nitride, which reduces plastic damage such as dislocations and lamination caused by mechanical stress. Meanwhile, compared with the TG process, the AlN substrate’s subsurface damage depth and surface roughness are reduced by 33% and 28.4%, respectively.

## Figures and Tables

**Table 1:** Parameters applied in the simulation model.

Machining Parameters	Value
Material structure	fibrillated zincite structure
Newtonian layer size (nm^3^)	18.1 × 10.8 × 7.0
Abrasive radius (nm)	3
Grinding depth (nm)	2
Grinding speed (m/s)	50
Vibration frequency (GHz)	0.1
Amplitude (nm)	0.8
Grinding distance (nm)	16
Laser heating temperature (K)	800
Initial temperature (K)	300
Potential function	Vashishta, Tersoff, Lennard-Jones
Relaxation system	NVT
Processing system	NVE

**Table 2:** The experimental items and grinding parameters [36].

Items	Laser Power (J/s)	Ultrasound Amplitude (µm)	Ultrasound Frequency (KHz)	Grinding Depth (mm)	Wheel Grit	Grinding Width (mm)	Feed Rate (mm/s)	Wheel Speed (rad/min)
1	-	-	-	0.02	320#	13	2	2500
2	500	-	-					
3	-	8	20					
4	500	8	20					

## Data Availability

The raw data supporting the conclusions of this article will be made available by the authors on request.
